# Mastication after craniotomy: pilot assessment of postoperative oral health-related quality of life

**DOI:** 10.1007/s00701-021-05020-w

**Published:** 2021-10-19

**Authors:** Mortimer Gierthmuehlen, Nadja Jarc, Dennis T. T. Plachta, Claudia Schmoor, Christian Scheiwe, Petra Christine Gierthmuehlen

**Affiliations:** 1 Department of Neurosurgery, University Medical Center Freiburg, Medical Faculty, Albert-Ludwigs-University Freiburg, Breisacher Strasse 64, 79106 Freiburg, Germany; 2 Neurosurgical Department, Ruhr-University Bochum, University Medical Center Knappschaftskrankenhaus Bochum, In der Schornau 23-25, 44892 Bochum, Germany; 3 Neuroloop GmbH, Engesser Str. 4, 79108 Freiburg, Germany; 4 Laboratory for Biomedical Microtechnology, Department for Microsystems Engineering (IMTEK), Albert-Ludwigs-University Freiburg, Georges-Koehler-Allee 102, 79110 Freiburg, Germany; 5 Clinical Trials Unit, Medical Centre-University of Freiburg, Faculty of Medicine, University of Freiburg, Freiburg, Germany; 6 Department of Prosthodontics, University Medical Center Duesseldorf, Moorenstraße 5, 40225 Duesseldorf, Germany

**Keywords:** OHIP, Craniotomy, Atrophy, QoL, Quality of life, Temporal muscle

## Abstract

**Background:**

Neurosurgical approaches to the brain often require the mobilization of the temporal muscle. Many patients complain of postoperative pain, atrophy, reduced mouth opening, and masticatory problems. Although the pterional, frontolateral-extended-pterional, and temporal craniotomies are the most frequently used approaches in neurosurgery, a systematic assessment of the postoperative oral health-related quality of life has never been performed so far. This study evaluates the oral health-related quality of life of patients after pterional, frontolateral-extended-pterional, or temporal craniotomy using a validated and standardized dental questionnaire, compares the results with the normal values of the general population, and investigates whether this questionnaire is sensitive to changes caused by surgical manipulation of the temporal muscle.

**Methods:**

The “Oral Health Impact Profile” (OHIP14) is a validated questionnaire to assess the oral health-related quality of life. It asks the patients to assess their oral health situation within the past 7 days in 14 questions. Possible answers range from 0 (never) to 4 (very often). Sixty patients with benign intracranial processes operated through a lateral cranial approach were included. The questionnaire was answered before surgery (baseline) and 3 months and 15 months after surgery.

**Results:**

Overall, postoperative OHIP scores increase significantly after 3 months and decrease after 15 months, but not to preoperative values. No factors can be identified which show a considerable relationship with the postoperative OHIP score.

**Conclusions:**

Postoperative impairment of mouth opening and pain during mastication can be observed 3 to 15 months after surgery and sometimes cause feedback from patients and their dentists. However, in line with existing literature, these complaints decrease with time. The study shows that the OHIP questionnaire is sensitive to changes caused by surgical manipulation of the temporal muscle and can therefore be used to investigate the influence of surgical techniques on postoperative complaints. Postoperatively, patients show worse OHIP scores than the general population, demonstrating that neurosurgical cranial approaches negatively influence the patient’s oral health-related wellbeing. Larger studies using the OHIP questionnaire should evaluate if postoperative physical therapy, speech therapy, or specialized rehabilitation devices can improve the masticatory impairment after craniotomy.

**Trial registration:**

Clinical trial register: DRKS00011096.

## Introduction

Several neurosurgical approaches require the incision of the temporal muscle (TM). Although the masseter muscle is the most important actor for mastication and jaw mobility, damage to the TM can have impact on mouth opening and chewing and may result in pain during movement. Additionally, the frequently observed postoperative atrophy can cause cosmetically unfavorable results leading to unhappy patients [2]. Personal communication (MG) with dentists and maxillofacial surgeons indicate that some patients contact their dentists after surgery as they experience difficulties with their mouth opening and pain in their temporomandibular joint. Interestingly, this problem has never been addressed by dentists using dental questionnaires and instruments examining oral health-related quality of life.

In the past, many authors have proposed modifications of the neurosurgical preparation technique in order to reduce both temporal muscle atrophy and impairment [3, 4, 9, 15, 18], but only few studies investigated postoperative complaints and atrophy with scientific, objective methods [1, 7, 10, 26, 27]. However, the questionnaires used in those studies were individually designed for the respective study and lacked both standardization and validation, as they did not compare the results with normal values of the general population. Furthermore, little is known about the postoperative course of these complaints and whether they disappear after a certain time.

In this prospective study, we applied the German version of the “Oral Health Impact Profile 14” (OHIP-G14), one of the most frequently used and validated questionnaires in dental research investigating oral health-related quality of life [25], to patients who were operated through a frontolateral-pterional, pterional, or temporal approach.

## Materials and methods

### Patient population

The study was approved by the local ethics committee of the university and registered at the clinical trials registry. All patients gave written informed consent. Between 2016 and 2019, we included 60 patients with a benign intracranial pathology (epilepsy, meningioma, aneurysm, orbital decompression) who were operated through a frontolateral-pterional, pterional, or temporal approach at our University Medical Center. Inclusion and exclusion criteria are described in Tables 1 and 2. The patients were asked by a doctor if they wanted to participate in the study, and the questionnaires were filled out by the patients. The study nurse kept track of all questionnaires. The survey was conducted in adherence to the good practice standard [14].

**Table 1:** Inclusion criteria of the OHIP study

Benign intracranial pathology
Intervention through a frontolateral-pterional, pterional, or temporal approach
Age > 18y

**Table 2:** Exclusion criteria of the OHIP study

Preoperative	Prior cranial surgery
	Hemiparesis
	Oral (mandibular pathology impairing mastication
	Malignant intracranial pathology
	Complete dentures
	Dementia/coma
Postoperative (persisting after 3 months)	Facial paresis > House and Brackman III°
	Neurological deficit impairing mastication
	Oral/mandibular pathology impairing mastication
	Second neurosurgical approach from contralateral side
	Anosmia

### Questionnaire

After informed consent to participate in the study, the German version of the OHIP-14 questionnaire (OHIP-G14, English version see Table 3) was filled out by the patients before surgery, 3 months after surgery, and 15 months after surgery. In order to increase adherence to the study, we chose 15 months, as the first postoperative visit in our outpatient clinic took place 3 months after surgery and the second visit 12 months later. Whenever a patient was not able to appear to the outpatient clinic in person, he/she received the questionnaire by mail and was asked to return it to our department.

**Table 3:** English version of the OHIP-14 questionnaire—in this study, we used the German version OHIP G14

For the past 7 days, have you…
…had trouble pronouncing any words because of problems with your teeth or mouth?
…felt that your sense of taste has worsened because of problems with your teeth or mouth?
…had painful aching in your mouth?
…found it uncomfortable to eat any foods because of problems with your teeth or mouth?
…been self-conscious because of your teeth or mouth?
…felt tense because of problems with your teeth or mouth?
…had to interrupt meals because of problems with your teeth or mouth?
…found it difficult to relax because of problems with your teeth or mouth?
…been a bit embarrassed because of problems with your teeth or mouth?
…been a bit irritable with other people because of problems with your teeth or mouth?
…had difficulty doing your usual jobs because of problems with your teeth or mouth?
…felt that life in general was less satisfying because of problems with your teeth or mouth
…been totally unable to function because of problems with your teeth or mouth?
Has been your diet been unsatisfactory because of problems with your teeth of mouth?

The OHIP-G14 consists of 14 questions overviewing the oral health-related quality of life (OHR-QoL) over the last 7 days. The questions can be answered from “never” to “very often,” and the respective answer is given a value from 0 (never) to 4 (very often). The values are then summarized and can therefore range from 0 to 56.

### Incision of the temporal muscle

**Fig. 1:**
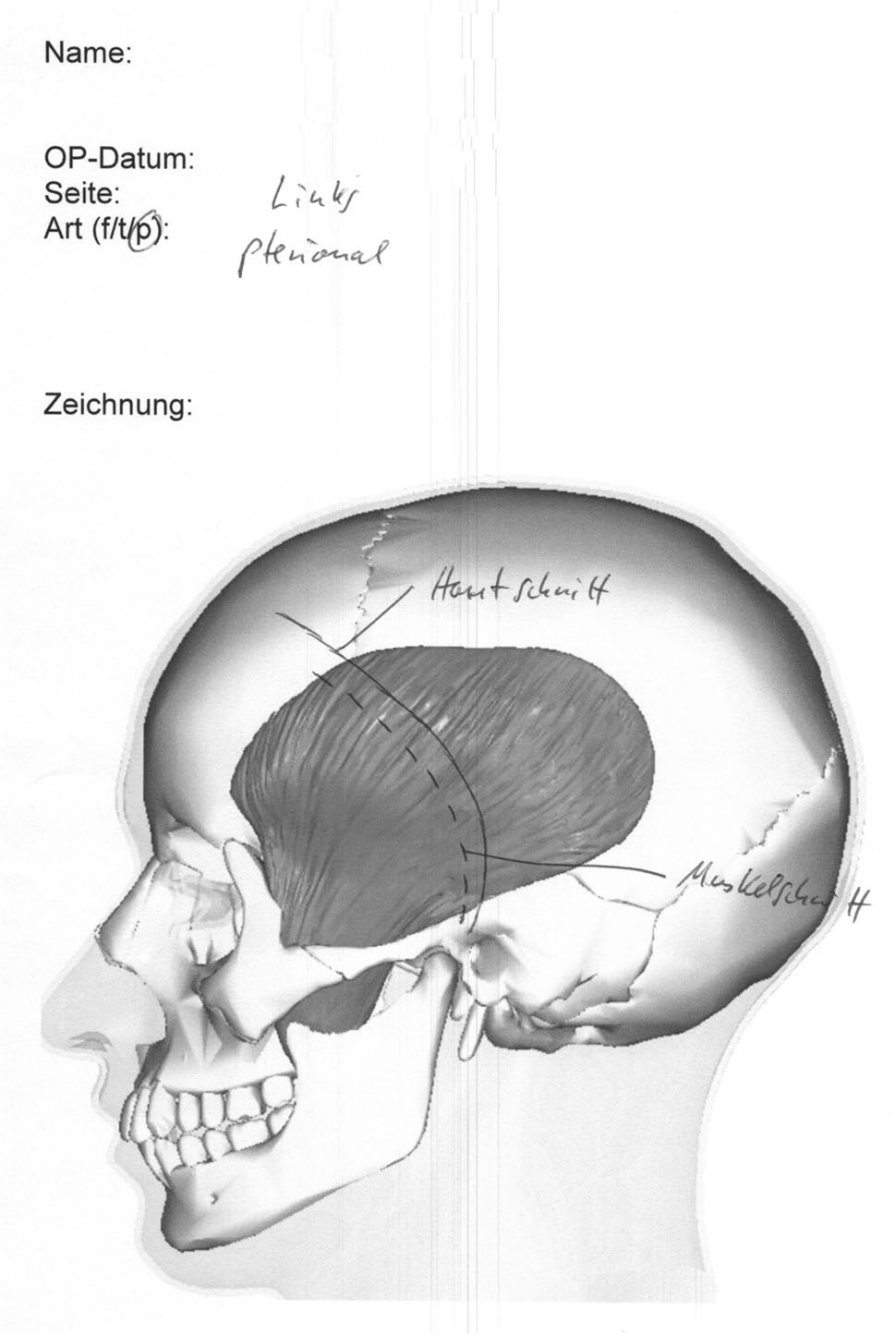
Example of the template in which the surgeons drew incision of the temporal muscle. Template model by BodyParts 3D DBCLS, licensed under CC 2.1 (http://lifesciencedb.jp/bp3d/)

After the operation, the surgeon was asked to draw the extent of TM incision on a printed picture of the temporal muscle (Fig. 1). This drawing was scanned and analyzed with FIJI (ImageJ v.2.1./1.53c, NIH, Bethesda, MD). The size of the muscle and the mobilized area with the zygoma being the caudal border were measured by the investigators and calculated using the “area” function.

### Statistical analysis

This was a single-center non-interventional study with the aim to investigate the change in oral health-related quality of life of patients who were operated through a frontolateral-pterional, pterional, or temporal approach. The primary endpoint was the difference between the OHIP-14 score 3 months post-surgery versus pre-surgery. The secondary endpoint was the difference between the OHIP-14 score 15 months post-surgery versus pre-surgery. Additionally, it was investigated if the change on OHIP-14 differed depending on the baseline characteristics sex (male vs female), age (< 50 vs 50 to < 60 vs ≥ 60 years), surgical approach (f vs p vs t), side (left vs right), and TM mobilization (< 30 vs 30 to < 40 vs ≥ 40).

Linear models for repeated measurements were used for statistical analysis (PROC MIXED, Statistical Analysis System Version 9.4). Mean OHIP-14 sum scores at the different visits and differences in OPHIP-14 sum scores between visits were calculated with 95% confidence intervals (CI). The statistical test of the hypothesis that the difference between the OHIP-14 sum score 3 months post-surgery versus pre-surgery (primary endpoint) is zero was performed at a two-sided significance level of 0.05. Statistical tests regarding secondary endpoints and additional objectives were conducted for exploratory purposes without alpha adjustment, and resulting *p*-values have to be interpreted in a descriptive sense.

## Results

Fifty-five patients entered the study, 14 (25.5%) males and 41 (74.5%) females. Thirty patients (54.5%) were operated on the left and 25 (45.5%) on the right side. In 6 patients (11%), the surgical drawing is missing and could not be reproduced, and 9 patients (16.3%) could not be reached after 15 months to fill out their final questionnaire. Seven patients (12.7%) had partial dentures, 44 patients (80%) had all-natural teeth, and 4 patients (7%) did not want to answer this question. The baseline characteristics are shown in Table 4. The mean size of TM incision was 37% (SD 16.38).

**Table 4:** Baseline characteristics of patient population. *m* male, *fe* female, *l* left, *r* right, *fr* frontolateral-pterional, *p* pterional, *t* temporal, *v* vascular, *od* orbital decompression, *me* meningioma, *e* epilepsy, *STD* standard deviation

Descriptive statistics				
Sex	m = 14 (25.5%)	fe = 41 (74.5%)	*n* = 55	
Age	Mean = 54.4	STD = 12.4	Range = 29–86	
Side	l = 30 (54.5%)	r = 25 (45.4%)		
Approach	fr = 20 (36.4%)	p = 26 (47.3)	t = 9 (16.3%)	
Diagnosis	v = 15 (27.3%)	od = 2 (3.6%)	me = 31(56.4%)	e = 7 (12.7%)
TM mobilization	Mean = 37.0%	STD = 16.4		

**Fig. 2:**
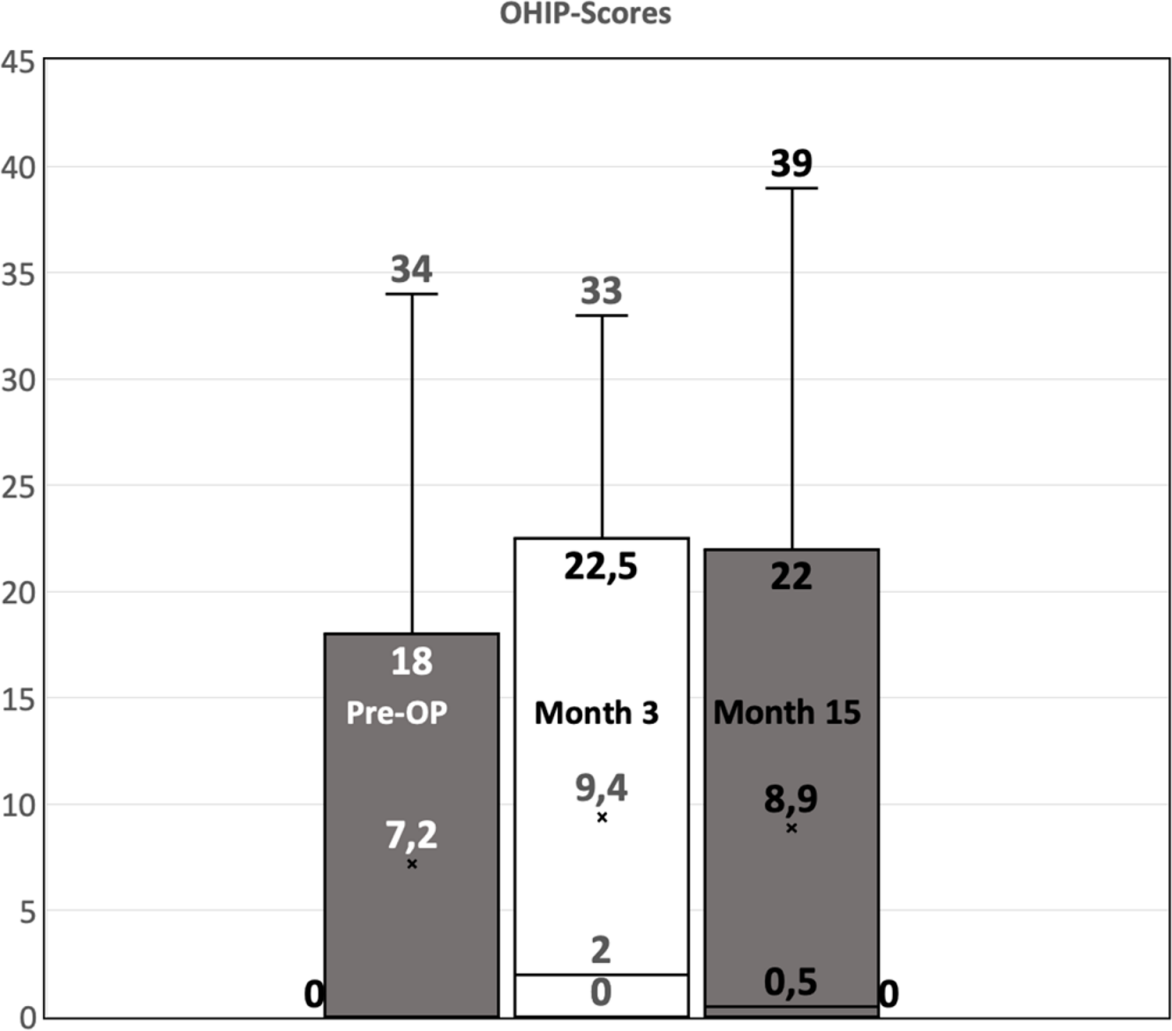
Boxplot of the OHIP scores at baseline, 3 months after surgery, and 15 months after surgery

Preoperatively, 48 patients (87.27%) had a sum of 4 and lower in the OHIP-G14 with 38 patients (69.09%) scoring 0. The baseline mean sum was 1.98 (95% CI 0.59–3.37). The development of the OHIP-14 sum score from pre-surgery to the post-surgery visits is shown in Fig. 2.

Three months after surgery (month 3), 31 patients (56.36%) had a sum score of 4 and lower with 21 patients (38.18%) scoring 0. The 3-month postoperative mean sum was 6.75 (95% CI 4.35–9.14). Seventeen patients (30.91%) had the same score as before surgery, 9 (16.36%) improved at least one point, and 29 (52.73%) worsened at least one point. The mean difference between the OHIP-14 sum score 3 months post-surgery versus pre-surgery was 4.76 (95% CI 2.27–7.264, *p* = 0.00034, Table 5), showing a significant decrease in oral health-related quality of life of patients 3 months post-surgery as compared to pre-surgery.

**Table 5:** Statistical analysis of the difference of preoperative and postoperative OHIP scores

Comparison of visits	Difference	95% CI lower limit	95% CI upper limit	*p*-value
Month 3 vs pre-surgery	4.76	2.27	7.26	0.00034
Month 15 vs pre-surgery	2.62	− 0.01	5.24	0.0504
Month 15 vs month 3	− 2.15	− 4.05	− 0.24	0.0282

Fifteen months after surgery (month 15), 33 (71.74%) showed a sum score of 4 and lower with 50% having 0 point. The 15-month postoperative mean sum was 4.60 (95% CI 2.17–7.03). Compared to the preoperative condition, 16 (34.78%) had the same score as before surgery, 11 (23.91%) were at least 2 points better than before surgery, and 19 (41.3%) were at least 1 point worse than preoperatively. The mean difference between the OHIP-14 sum score 15 months post-surgery versus pre-surgery was 2.62 (95% CI − 0.01 to 5.24, *p* = 0.0504). The mean difference between the OHIP-14 sum score 15 months post-surgery versus 3 months post-surgery was − 2.15 (95% CI − 4.05 to − 0.24, *p* = 0.0282, Table 5). This indicated that the oral health-related quality of life of patients improved from 3 to 15 months post-surgery. Ten of the patients were operated with a myocutaneous flap and showed a mean OHIP score of 1 preoperatively, which changed to 6.1 after 3 months and to 4.5 after 15 months. The remainder (*n* = 45) were operated with two-layer preparation and had a preoperative OHIP score of 2.1, which changed to 6.6 after 3 months and to 5.1 after 15 months.

The differences between the post-surgery OHIP-14 sum scores and the pre-surgery scores dependent on the baseline characteristics sex, age, surgical approach, side, and the amount of TM mobilization are shown in Table 6. None of these factors showed a considerable impact on the postoperative OHIP-G14 results. For TM mobilization, the largest difference was seen for the mid-category 30 to < 40, but there was no trend with increasing amount.

**Table 6:** Statistical analysis of the difference of preoperative and postoperative OHIP scores dependent on baseline characteristics. For each factor, a separate linear model for repeated measurements was used with adjustment for the pre-surgery OHIP-14 sum score

Factor	Value	Visit	Difference to pre-surgery	95%-CI lower limit	95%-CI upper limit	*p*-value (effect of factor)	*p*-value (interactive effect between factor and visit)
Sex	Male	Month 3	2.32	− 2.34	6.99	0.47	0.24
	Female	Month 3	5.46	2.74	8.19		
	Male	Month 15	2.04	− 2.82	6.90		
	Female	Month 15	2.61	− 0.25	5.47		
Age	< 50	Month 3	5.35	1.05	9.64	0.73	0.25
	50– < 60	Month 3	5.31	1.59	9.03	-	
	≥ 60	Month 3	2.91	− 1.72	7.53	-	
	< 50	Month 15	0.78	− 3.63	5.19		
	50– < 60	Month 15	3.88	0.07	7.69	-	
	≥ 60	Month 15	2.21	− 2.49	6.91	-	
Approach	fr	Month 3	5.32	1.44	9.20	0.18	0.26
	p	Month 3	5.86	2.48	9.24		
	t	Month 3	− 0.24	− 5.98	5.51		
	fr	Month 15	4.74	0.77	8.70		
	p	Month 15	1.94	− 1.67	5.55		
	t	Month 15	− 1.56	− 7.45	4.33		
Side	Left	Month 3	5.01	1.78	8.23	0.68	0.86
	Right	Month 3	4.25	0.72	7.78		
	Left	Month 15	3.00	− 0.37	6.36	-	
	Right	Month 15	1.90	− 1.69	5.49	-	
Mobilization	< 30	Month 3	2.10	− 3.05	7.26	0.051*	0.59
	30– < 40	Month 3	8.01	3.66	12.37	-	
	≥ 40	Month 3	3.50	− 0.50	7.50	-	
	< 30	Month 15	− 1.64	− 6.71	3.43		
	30– < 40	Month 15	7.04	2.63	11.44	-	
	≥ 40	Month 15	1.37	− 2.72	5.46	-	

^*^Test for difference between categories: *p* = 0.051, test for trend: *p* = 0.83

## Discussion

### Main findings

The mean baseline OHIP score of the patients in our study was within the range of the normal population [12]. Three months after surgery, the mean is significantly worse and exceeds the range of the general population. The “minimal important difference” (MID) in OHIP-14 scores differs in literature and ranges from 3 [19] to 5 [16]. In our study, the mean difference was 4.76, which in our cohort was already significant. After 15 months, the score is still above the threshold of the general population but tends to decrease. None of the investigated baseline factors had a considerable influence on the postoperative OHIP score, neither had the percentual size of TM incision. The preparation technique “myocutaneous” vs “two-layer preparation” shows a tendency towards better results in the myocutaneous flap, but with *n* = 10 in the myocutaneous group, this is not significant. This tendency is in line with a study by Andrade et al. which observed that interfascicular preparation caused significantly longer-lasting pain than myocutaneous flap preparation [1].

Both after 3 and 15 months, some patients showed better scores than before surgery (Fig. 2). At first, this seems illogical but could be interpreted that stress before a neurosurgical intervention might negatively influence the results of the questionnaire. Additionally, the increased focus on the wound combined with the temporary postoperative application of analgetic medication could have an impact on general oral health-related QoL. In line with the results of Yasuda and Costa [7, 27], the postoperative impairment of the oral health-related QoL decreases with time. With a 3-month postoperative mean of 6.75, the oral impairment after cranial surgery is still below the mean observed in adolescents after previous dental trauma in childhood [5] and in patients with mandibular disc displacement [17]. This study shows that the score of the OHIP questionnaire is influenced by neurosurgical interventions and causes an increase of the score above normal values of the general population. In larger studies, OHIP can therefore be used to quantify oral health-related impairment of neurosurgical procedures with respect to surgical techniques and postoperative care.

### Limitations of the study

With 60 patients, a robust subgroup analysis is not possible regarding age, sex, side of surgery, and mobilization as well as type of approach. For these questions, a larger patient population is needed. Also, we included all 6 board-certified neurosurgeons of our department with different levels of skills (between 5 and 30 years after residency) and slightly different preparation techniques of the TM. With the inclusion and exclusion criteria applied (Table 1), it took 3 years to complete the study. Focusing on only one or two neurosurgeons would have increased the time needed for the study tremendously. The major downside of this study is the fact that we did not evaluate the preoperative and postoperative dental status of each patient each time the questionnaire was given to the patient. We did that on purpose as (1) such investigation would have increased the stress to the patient before and after surgery, (2) the compliance of the patients would have been less, and (3) the patients’ oral status includes so many criteria that defining “dentally” comparable groups would have been extremely difficult. It can of course be criticized that during a period of 15 months, other dental factors could influence the outcome of the OHIP questionnaire.

### Relation of the present study to previous work

Many neurosurgical approaches to the skull base, to the frontal, and to the temporal lobe require the more or less extensive incision and mobilization of the temporal muscle. Postoperative scarring and pain can lead to impairment of mastication and to a limited mouth opening, and further atrophy can cause cosmetically unfavorable results [2]. Different modifications of the surgical approaches have been proposed in the past to address muscular atrophy, including suturing the muscle to holes or screws in the temporal bone [3, 4] and inserting implants to reconstruct the shape of the former muscle [15]. However, these publications used arbitrary and non-standardized questions regarding postoperative complaints and did not investigate the actual impairment of the patients with objective methods or questionnaires. This point is addressed by the present study which uses a standardized questionnaire, and to our knowledge, our study is the first applying the OHIP-G14 in neurosurgical patients.

Systematic and standardized studies regarding the postoperative impairment of the TM are rare. Andrade et al. investigated 68 patients after aneurysm clipping which were divided into two groups with either myocutaneous flap or intrafascicular preparation of the TM. They investigated cranial nerve function and dental parameters (temporomandibular joint (TMJ) and occlusion by a dentist) after 180 days, but the scales they used were not standardized [1]. Also Welling et al. investigated the subjective impairment after larger or smaller craniotomy for aneurysm clipping [26]. More objective parameters were investigated by Yasuda et al. in 2010, who observed a significant reduction of TM volume in MR volumetry and a nearly halving of EMG activity on the operated side. They also found a limited mouth opening after surgery which improved over time [27]. Another MR volumetric study was performed by Hwang et al. in 2010 who found no difference in atrophy whether monopolar preparation was used or not [10]. This goes in line with our observation that the percentual size of TM incision does not significantly correlate with an increased OHIP score, though a tendency is visible that < 30% mobilization might be beneficial. A larger study with focus on TM incision, preparation method, and its impact on OHIP might clarify this aspect. One of the most extensive dental investigations was performed in the study by Costa et al. in 2014, who examined 24 patients after epilepsy surgery 6 and 12 months after surgery. They observed that mouth opening was limited after surgery and that patients complained of TMJ pain and sounds. As in our study, these symptoms decreased over time, but it was shown that preoperative bruxism was a predictor for worse outcome.

Besides objective parameters like mouth opening, range of motion, and bite force, oral health is often very subjective, and its assessment and emphasis on quality of life (QoL) differs from patient to patient. Therefore, the Oral Health Impact Profile (OHIP) with 49 questions was proposed by Slade and Spencer in 1994 [25]. As the compliance to fill out questionnaires recedes with the number of questions, a shorter version with 14 questions was introduced and evaluated by Slade in 1997 with a comparable reliability to the longer version [24]. The questionnaire was translated to German (OHIP-G14) by John et al. in 2002 [13]. In 2004 this group defined reference values for OHIP-G14 in the normal population [12]. With partial dentures, 50% of the population has a sum score of 4 and lower.

Usually, the OHIP questionnaire is applied in longitudinal studies to evaluate a dental intervention [6, 11]. Realizing the massive impact of oral health on the general QoL, OHIP has recently been used to investigate QoL after transplantation [21–23] and other medical conditions [8, 20]. This study adds the knowledge that also neurosurgical interventions can negatively influence oral health-related quality of life and that this influence can be detected by a standardized dental questionnaire. OHIP is therefore a tool which can be used to detect the influence of surgical preparation techniques. It could also be used as a tool to investigate whether specific physical therapy, e.g., speech therapy, or special rehabilitation devices are suitable to improve the postoperative, oral health-related complaints.

## Conclusion

Neurosurgical approaches through the temporal muscle cause significant postoperative impairment of the oral health-related quality of life, which can be detected by a well-known, standardized, and validated dental questionnaire.

In this study, no factors can be identified which show a considerable impact on the level of impairment. After 15 months, the impairment tends to decrease but does not reach baseline values.

The OHIP questionnaire is a powerful tool which helps to objectify and quantify oral complaints after neurosurgical interventions. It shall be used in larger and longer studies to investigate whether surgical preparation techniques, physical therapy, speech therapy, or specialized rehabilitation devices (e.g., the Rehabite® system) can improve the oral health-related impairment after craniotomy.
